# Effect of Different Drying Techniques on the Bioactive Compounds, Antioxidant Ability, Sensory and Volatile Flavor Compounds of Mulberry

**DOI:** 10.3390/foods13162492

**Published:** 2024-08-08

**Authors:** Jing Zhang, Jing Chen, Jingsha Lan, Bingliang Liu, Xinhui Wang, Suyi Zhang, Yong Zuo

**Affiliations:** 1 College of Food and Biological Engineering, Chengdu University, Chengdu 610106, China; zhangjing2022@cdu.edu.cn (J.Z.); 18381307702@163.com (J.C.); liubingliang@cdu.edu.cn (B.L.); 2 Luzhoulaojiao Postdoctoral Programme Luzhou Laojiao Co., Ltd., Luzhou 646000, China; zhangsy@lzlj.com; 3 College of Life Science, Sichuan Normal University, Chengdu 610101, China; lanjingsha@outlook.com; 4 Chengdu National Agricultural Science and Technology Center, Chengdu 610213, China; 5 Luzhou Laojiao Co., Ltd., Luzhou 646000, China

**Keywords:** mulberry, drying methods, bioactive ingredients, drying curve, sensory evaluation

## Abstract

Mulberry perishes easily due to its high water content and thin skin. It is important to extend the shelf life of mulberry by proper processing methods. In the present study, the influence of three drying techniques, including hot air drying (HAD), vacuum drying (VD), and vacuum freeze-drying (VFD) on the quality maintenance of mulberry was comprehensively evaluated. Bioactive compounds, antioxidant activity, and the sensory and volatile flavor compounds of mulberry have been researched. The results showed that VFD treatment maintained the highest anthocyanins (6.99 mg/g), total flavones (3.18 mg/g), and soluble sugars (2.94 mg/g), and exhibited the best DPPH· (81.2%) and ABTS^+^· (79.9%) scavenging ability. Mulberry also presented the lowest hardness and the greatest brittleness after VFD. Additionally, VFD maintained the optimal color and presented the best sensory attributes. Furthermore, 30, 20, and 32 kinds of volatile flavor compounds were detected in HAD, VD, and VFD, respectively, among which aldehydes, esters, and ketones were the most abundant compounds. This study indicated the potential application value of VFD for the drying of fruit and vegetable foodstuffs.

## 1. Introduction

Mulberry, the mature fruit of *Morus alba*, is rich in nutrients: vitamins, amino acids, minerals, and the bioactive compounds of anthocyanins, polysaccharides, and flavonoids, exhibiting the functions of liver protection, blood sugar reduction, and anti-aging, anti-inflammation, anti-mutation, anti-tumor, and immune regulation activities [1,2]. Mulberry can not only be eaten directly but can also be processed into fruit wine, fruit vinegar, and other products, exhibiting great economic value. However, mulberry is a kind of high-water content fruit with the characteristics of short-term maturity and thin skin; it is fragile, deteriorates easily, and is intolerant of storage and transportation, which leads to a waste of resources and inhibits the healthy development of the mulberry industry. Thus, it is necessary to take proper methods for the deep processing of mulberry. Drying is an effective method of deep processing that can reduce losses in the transportation and storage process and has been widely applied in fruit and vegetable food processing [3].

At present, the fruit and vegetable food drying process mainly depends on hot air drying (HAD), vacuum drying (VD), and vacuum freeze-drying (VFD) [4]. HAD presents the advantages of being simple, fast, and highly efficient. However, it is easy to cause surface shrinkage, hardening, and nutrient loss in the products [5]. VD operates under low-pressure conditions, with the advantages of speed and stable quality [6]. It also faces the drawbacks of high cost and complex operation. VFD can effectively remove water in food, based on the principle of solid ice crystal sublimation, which can produce loose pores on the surface of the material. However, the product requires a long time to dry [7].

During the drying process, the product’s nutrients, antioxidant activity, appearance, and color may change. In Chen’s research, freeze-explosion puffing drying (F-EPD) proved to be a good way to maintain the antioxidant capacity of mulberry [7]. According to Michalska’s research, when processed with FD, the antioxidant capacity of blackcurrant declined [8]. Additionally, drying also changes the color and hardness of food, affecting the taste and quality of products [9]. However, the effect of drying on volatile flavor compounds is not clear. Therefore, choosing the appropriate drying method to reduce the loss of nutrients and flavor components in mulberry has become an important topic in drying process research.

**Figure 1:**
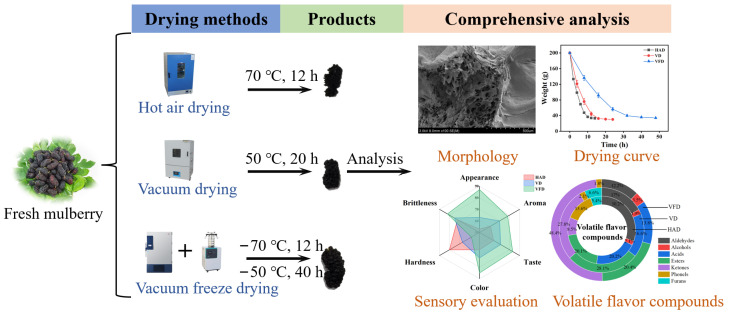
The schematic of mulberry drying.

In the present study, HAD, VD, and VFD were applied for the drying of mulberry (Figure 1). By comparing the effect of three drying methods on the physicochemical properties and volatile flavor compounds of mulberry, the optimal drying method of VFD was determined. This research exhibits strategic significance for improving the technical level of mulberry processing and for promoting the development and utilization of mulberry resources.

## 2. Material and Methods

### 2.1. Raw Material and Reagents

Fresh mulberry was purchased from Yibin, Sichuan Province, China, and was stored at −20 °C for further analysis.

Sodium hydroxide, sodium carbonate, ethanol, potassium chloride, sodium nitrite, aluminum nitrate, glucose, rutin, gallic acid, DPPH· and ABTS^+^·were obtained from Sinopharm Chemical Reagent (Shanghai, China). All reagents used in the assays were of at least analytical grade and were used without further purification.

### 2.2. Drying Procedures

HAD: the mulberry was evenly spread out and put into the air-blast-drying oven (GZX-9000 MBE, Shanghai Boxun Medical Biological Instrument Co., Ltd., Shanghai, China). The drying temperature was set to 70 °C. The mulberry was weighed every 2 h until it reached a constant weight.

VD: the mulberry was evenly spread out and put into a vacuum-drying oven (DZF-6000 MBE, Shanghai Boxun Medical Biological Instrument Co., Ltd., Shanghai, China). The drying temperature was set to 50 °C. The mulberry was weighed every 4 h until it reached a constant weight.

VFD: first, the mulberry was put into a constant-temperature freezer (BCD-539WT, Haier, Qingdao, China). The temperature was set to −70 °C and held for 12 h. Then, the mulberry was put into the vacuum freezer (SCIENR2-10N1C, Ningbo Xinzhi Biotechnology Co., Ltd., Ningbo, China) with a vacuum degree below 50 Pa and a cold trap temperature of −50 °C. The mulberry was weighed every 8 h until it reached a constant weight.

The drying methods and conditions are presented in Table 1. The dried mulberry was first ground and sealed, then stored in a refrigerator at 4 °C.

### 2.3. Reducing Sugar and Soluble Solids

Reducing sugar was detected using the 3,5-dinitrosalicylic acid (DNS) method [10]. Soluble solids were detected by referring Burdon’s report [11].

### 2.4. Moisture Content

Moisture content was determined according to Köprüalan’s research, with slight modifications [12]. Briefly, a 5 g mulberry sample was added into a weighing bottle and dried in an oven at 105 °C. The sample was weighed every two hours until it reached a constant weight. The moisture content was calculated according to the formula:

X = (m1 − m2)/(m1 − m3) × 100

where X: moisture content (%); m1: the total mass of the weighing bottle and sample (g); m2: the total mass of the weighing bottle and dried sample (g); m3: the mass of the weighing bottle (g).

### 2.5. Bioactive Compounds and Antioxidant Ability

Anthocyanins were detected using the pH differential method according to Cheng’s research, with slight modifications [13]. First, a 0.5 g sample was added to 10 mL ethanol-HCl solution (6 mL ethanol (95%) and 4 mL HCl (1%)). After a water bath at 40 °C for 2 h and centrifugation at 8000 r/min for 2 min, 2 mL of supernatant was introduced into 23 mL pH 1.0 and pH 4.5 buffer solutions, respectively. After 15 min of reaction, the absorption at 520 nm and 700 nm were measured, respectively. Anthocyanin content was calculated according to the following formula:

A = (A520–A700) pH1.0 − (A510–A700) pH4.5

C = (A × Mw × Df)/(ε × 1)

C is the anthocyanin content (mg/g). (A510–A700) pH1.0 is the difference in the sample absorption value in the presence of pH 1.0 buffer. (A510–A700) pH4.5 is the difference in sample absorption value in the presence of a pH 4.5 buffer. Mw is the molecular weight of cyanidin-3-glucoside, 449.2. Df is the dilution ratio; ε is the molar extinction coefficient of cyanidin-3-glucoside, 29,600.

Total flavones were detected according to Borah’s research, with slight modifications [14].

For this purpose, 1 g of dried mulberry sample was added to 30 mL ethanol (60%) and the solution was treated with ultrasound for 10 min. After centrifugation at 8000 r/min for 2 min, the supernatant was obtained.

Total flavones: 5 mL of supernatant was mixed with 2 mL NaNO_2_ (5%). After 5 min, 2 mL Al (NO_3_)_3_ (10%) was added, and the solution was incubated for 6 min. Then, 20 mL NaOH (1 mol/L) and 21 mL ethanol (30%) were further introduced. After 15 min of reaction, the absorbance at 510 nm was measured. The standard curve of rutin was drawn and the results are expressed in units of mg/g rutin.

Phenols were determined with a high-performance liquid chromatograph triple quadrupole mass spectrometer (LCMS-8050, SHIMADZU, Kyoto, Japan).

Sample preparation: 1 g of mulberry sample was weighed and added into 20 mL methanol. After ultrasound treatment for 30 min, the solution was obtained by passing it through a 0.22 µm filter membrane. 

GC condition: This was tested with a chromatographic column, the Leapsil C18 (2.7 µm × 100 × 2.1 mm). The mobile phase was composed of 0.1% formic acid (phase A) and 100% acetonitrile (phase B), and the flow rate was 0.2 mL/min. The injection volume was 1 µL. The column temperature was 40 °C. The following elution gradients were used: 0.0–2.0 min, 100% A; 2.0–5.0 min, 90% A; 5.0–8.1 min, 10% A; 8.1–12.0 min, 90% A.

MS condition: The MS test was conducted using electrospray ionization (ESI). The samples were scanned simultaneously in positive/negative ion mode and detected in the multiple reaction-monitoring (MRM) mode. The ion source temperature was 300 °C. The electrospray voltage was 4000 V. The flow rates of the curtain gas, atomization gas, and auxiliary gas were 10, 3, and 10 L/min. The pressure of the collision gas was 270 kPa.

The quantitative analysis of phenols was carried out according to the external standard method.

DPPH· scavenging rate and ABTS^+^· scavenging rate were detected according to Xu’s research [15], and their units were expressed as a percentage.

### 2.6. Hardness and Brittleness 

Hardness and brittleness were measured with a texture analyzer (Texture Technologies Corp., New York, NY, USA). The test speed was 30 mm/min, and the speed before and after the test was 30 and 50 mm/min, respectively. The triggering force was 1 N, and the detection distance was 8 mm. The dried mulberry test was repeated five times.

### 2.7. Color

The color was measured with an automatic color difference meter (UltraScan VIS, HunterLab, Reston, VA, USA). It was expressed using the color difference value (Δ*E*):

$$\Delta E=\sqrt{{\Delta L}^{2}+{\Delta a}^{2}+{\Delta b}^{2}}$$

*L* is the brightness index, and *a* and *b* are indicators of the color chroma. Δ*L*, Δ*a*, and Δ*b* are the difference in the *L*, *a*, and *b* values of mulberry before and after drying. 

### 2.8. Microstructure of Mulberry

The microstructure of mulberry was characterized using a scanning electron microscope (Quattro, Thermo Fisher Scientific Co., Ltd., Waltham, MA, USA).

### 2.9. Volatile Flavor Compounds

For the headspace solid phase microextraction (HS-SPME), 2 g dried mulberry powder was introduced into a headspace bottle containing 2 mL NaCl solution (0.2 g/mL) and 10 µL 2-octanol (1 g/L). The bottle was heated at 45 °C for 30 min. The extraction head was inserted into the headspace bottle for 40 min.

GC/MS analysis was carried out on the GCMS-QP2020NX device (Shimadzu, Japan). The chromatographic separation was performed on the HP-INNOWAX (60 m × 0.25 µm, 0.25 mm). The temperature procedure was divided into 4 stages: (1) kept at 40 °C for 30 min; (2) increased to 120 °C at a rate of 5 °C/min; (3) increased to 240 °C at a rate of 8 °C/min and held for 5 min; (4) increased to 250 °C at a rate of 5 °C/min and held for 1 min. The carrier gas was highly pure He (99.999%), with a flow rate of 1 mL/min and no split flow. The MS analysis was conducted in electron ionization (EI) at 70 eV. The ion source temperature was 230 °C. The interface temperature was 250 °C.

Each compound was determined by comparing the NIST database library with matching degrees of >80%. Compounds were semi-quantitatively analyzed with the internal standard method, using 2-octanol as the internal standard.

### 2.10. Sensory Evaluation

For the sensory evaluation, 30 trained testers were randomly selected to evaluate the appearance, aroma, taste, color, hardness, and crispness of mulberry samples. The sensory scoring was classified as follows: 10–20, dislike very much; 30–40, dislike moderately; 50–60, neither like nor dislike; 70–80, like moderately; 90–100, like very much.

### 2.11. Statistical Analysis

All experiments were repeated three times, and the results were expressed as mean ± standard deviations (SD). Partial least squares discriminant analysis (PLS-DA) was conducted using Simca 14.1.

## 3. Results and Discussions

### 3.1. Determination of Bioactive Compounds

**Figure 2:**
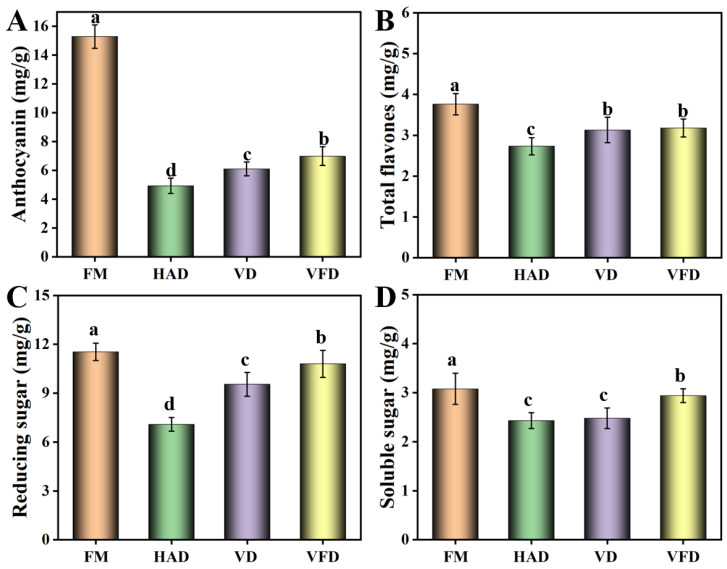
Effects of drying methods on the active ingredients in mulberry. (**A**) Anthocyanin. (**B**) Total flavones. (**C**) Reducing sugar. (**D**) Soluble sugar. FM: fresh mulberry. HAD: hot air drying. VD: vacuum drying. VFD: vacuum freeze-drying. The parameters were for DW. Different letters represented statistically different values at *p* < 0.05.

The bioactive compounds of anthocyanins and flavones in mulberry were analyzed (Figure 2). Anthocyanins, one of the most important compounds in mulberry, have anti-oxidant, anti-cancer, and anti-inflammation functions and can be applied as natural pigments, exhibiting broad prospects in the fields of food and medicine [16,17]. Compared with the values of fresh mulberry, after drying with different methods, the content of anthocyanins decreased to different degrees and the residual contents were less than 50% of that of fresh mulberry. Flavones, an important secondary metabolite during mulberry growth, exhibit anti-tumor and anti-bacterial capability [18]. When processed by different methods, the total flavones exhibited a modest decrease. With VD and VFD treatment, the total flavones maintained over 80% of the flavones of fresh mulberry. The reducing sugar value was also determined. Compared with fresh mulberry, the content of reducing sugar decreased to different degrees. Soluble solids, a class of water-soluble compounds mainly including soluble sugar, represent a technological parameter that is applied to evaluate mulberry maturity [19]. Soluble solids showed the highest value in VFD, followed by VD and HAD. During the process of VFD and VD treatments, pigment browning and sugar oxidation can be effectively avoided under low-oxygen conditions, leading to good retention of soluble solids [15]. However, when processed by HAD, the slow hydrolysis reaction and Maillard reaction decreased the contents of soluble solids [20]. Phenols, which are widely found in plants, are functional components with good antioxidant activity, exhibiting broad prospects in improving the quality of products [21]. The results indicated that protocatechuic acid and chlorogenic acid were relatively higher in terms of phenols in mulberry (Table 2). When dried by the different methods, their contents presented the phenomenon of increasing, which may be caused by the transformation of other phenols under the influence of high temperature. In addition, compared with fresh mulberry, the contents of gallic acid, ferulic acid, and cumaric acid declined to different degrees. According to Ashtiani’s study, the decrease in phenols seen during the drying process may be derived from the combination of phenols and proteins, and from the activation of polyphenol oxidase [22].

### 3.2. Antioxidant Activity

**Figure 3:**
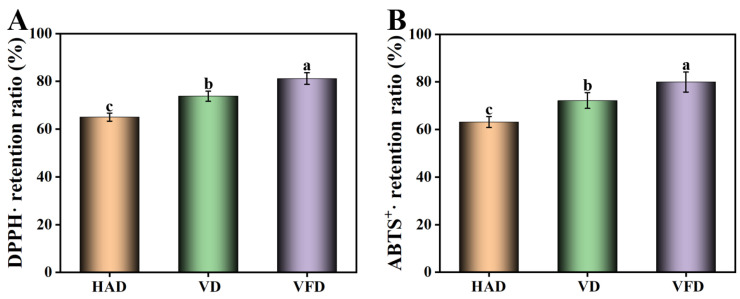
Effects of drying methods on the antioxidant activity of mulberry. (**A**) DPPH· retention ratio. (**B**) ABTS^+^· retention ratio. HAD: hot air drying. VD: vacuum drying. VFD: vacuum freeze-drying. Different letters represented statistically different values at *p* < 0.05.

Mulberry is rich in anthocyanins and flavones, which endow products with great antioxidant ability [23,24]. As the results show in Figure 3, the DPPH· scavenging ability of the different mulberry samples all maintained high levels. The retention ratios of the DPPH· scavenging capacity of mulberry dried by HAD, VD, and VFD were 65.03%, 73.80%, and 81.19%, respectively. According to Chan’s research, during the thermal drying process, the antioxidant ability declined more markedly than with non-thermal drying [25]. The ABTS^+^· scavenging ability has been also explored. After drying with different methods, all mulberry samples presented a scavenging ability of more than 60%.

### 3.3. Hardness and Brittleness

**Figure 4:**
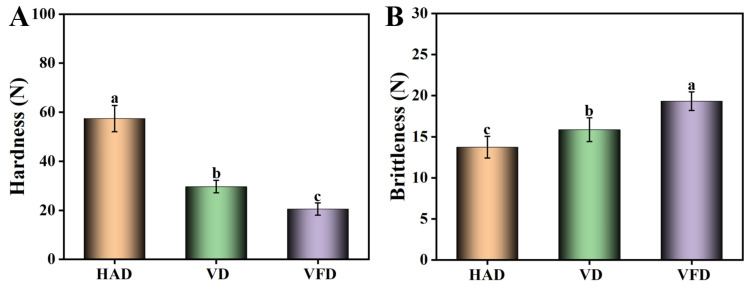
Effects of drying methods on the hardness and brittleness of mulberry. (**A**) Hardness. (**B**) Brittleness. HAD: hot air drying. VD: vacuum drying. VFD: vacuum freeze-drying. Different letters represented statistically different values at *p* < 0.05.

After drying with HAD, the mulberry samples presented the highest level of hardness and similar brittleness to those dried by VD (Figure 4). In contrast, mulberry samples dried by VFD exhibited the lowest level of hardness (20.5 N) and the highest level of brittleness (19.3 N). During the processes of HAD and VD, the high temperature affected the tissue structure of the mulberry and decreased the porosity, leading to a high level of hardness in the products [26]. In VFD, the ice crystallization directly sublimated and the pores were enlarged since the mulberry samples were exposed to a low-temperature and vacuum environment. Therefore, the fruit structure tended to be soft, although the hardness and brittleness levels were more suitable [27].

### 3.4. Color

Color is an important index when judging the quality of dried mulberry. As shown in Table 3, the *L*, *a*, *b*, and Δ*E* values of dried mulberry were different from each other. Compared with fresh mulberry, the *L*, *a*, and *b* values increased to varying degrees, which can be attributed to the increase in relative concentration of anthocyanins, owing to the massive loss of water [28]. Mulberry samples dried by VFD presented the highest *L*, *a*, *b*, and Δ*E* values, due to the pigment being effectively maintained in the vacuum environment. When dried by HAD, mulberry showed the lowest *L* and *b* values and a moderate *a* value, which may be caused by pigment degradation due to the high-temperature environment [29].

### 3.5. Drying Curve

**Figure 5:**
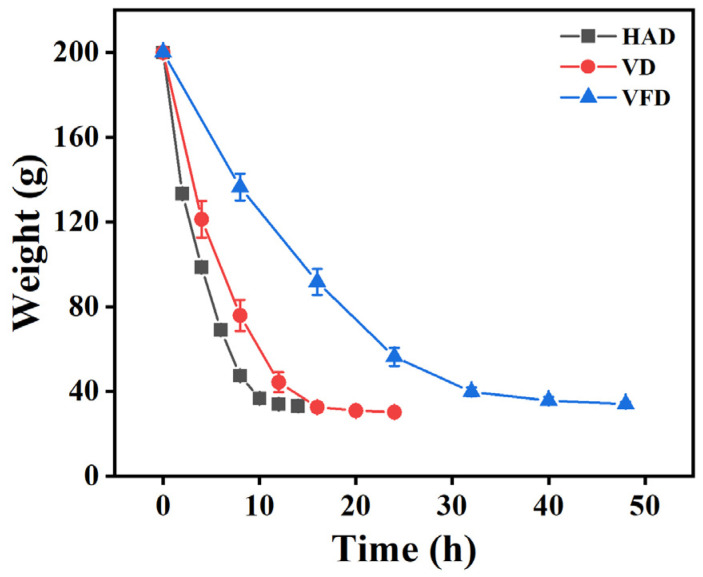
Drying curves of the HAD, VD, and VFD samples. HAD: hot air drying. VD: vacuum drying. VFD: vacuum freeze-drying.

The dynamic change in mulberry mass is shown in Figure 5. At the early stage of drying, the mass of the mulberry rapidly declined owing to the movement of water from the bigger capillaries. At the later stage, the drying rate decreased as water was removed from the smaller capillaries [30]. The order of mulberry mass after drying was VFD > HAD > VD. Among them, the mulberry treated by HAD decreased from 200 to 33.13 g after drying for 14 h, and there was no significant difference from 12 to 14 h, indicating that it was basically stable after drying for 12 h. With the VD and VFD samples, relatively long drying times of 20 and 40 h were required. After drying by different methods, the moisture content was 17.67, 18.75, and 19.61%, respectively, for HAD, VD, and VFD.

### 3.6. Microstructure of Dried Mulberry

**Figure 6:**
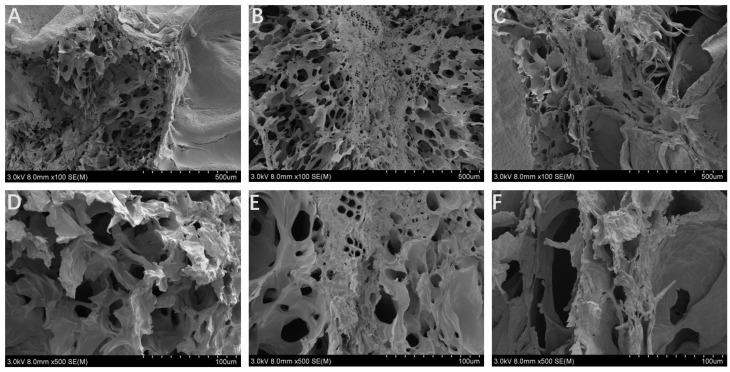
Effects of drying methods on the morphology of mulberry. (**A**,**D**) hot air drying (HAD); (**B**,**E**) vacuum drying (VD); (**C**,**F**) vacuum freeze-drying (VFD).

The microstructure of the mulberry samples was correlated with the different drying methods. The water evaporation of HAD and VD is derived from inward-to-outward diffusion. When dried by HAD, mulberry was affected by the high temperature for a long time, accompanied by serious damage to the tissue structure and increasing hardness (Figure 6). During the VD process, water was lost in the vacuum conditions, with the samples retaining the honeycomb hole structure. However, the external form shrank due to the loss of water. Much more dense structures were observed in the HAD and VD samples, which can be ascribed to the shrinkage and collapse of cells during the drying process [7]. In VFD, the moisture was sublimated directly after forming ice crystals under vacuum and low-temperature conditions, causing a loose and orderly microstructure and fluffy and full morphology, indicating that FD had a positive role in maintaining the porous cellular structure [31].

### 3.7. Sensory Evaluation

**Figure 7:**
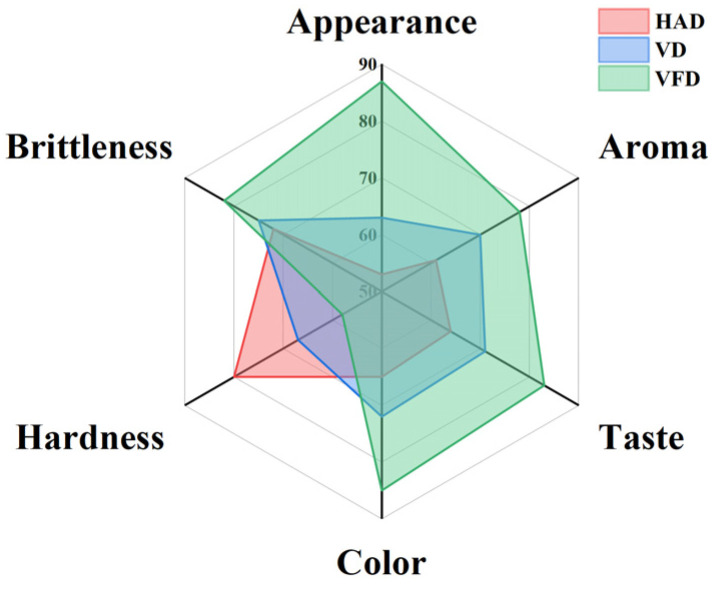
Sensory evaluation of mulberry samples dried by different methods. HAD: hot air drying. VD: vacuum drying. VFD: vacuum freeze-drying.

The mulberry samples were further analyzed by evaluating their appearance, aroma, taste, color, hardness, and brittleness. As the results in Figure 7 show, the VFD mulberry samples presented the best appearance, aroma, taste, color, and brittleness, and the lowest hardness. In contrast, when processed by HAD, the mulberry samples exhibited the highest level of hardness. When dried with VD, the sensory indexes were between the values for VFD and HAD.

### 3.8. Analysis of Volatile Flavor Compounds

During the process of drying, the volatile flavor components of mulberry may be lost owing to changes in temperature and pressure. As the results show in Table 4, a total of 47 volatile flavor components in mulberry were identified and quantified using HS-SPME-GC-MS, including 6 alcohols, 12 aldehydes, 6 acids, 13 esters, 3 ketones, 3 phenols, and 3 furans. As the results show in Figure S1 in the Supplementary Materials, 30, 20, and 32 kinds of volatile flavor compounds were detected in the mulberry samples. The difference in the volatile flavor compounds may be derived from the volatilization of low-volatile compounds and the interconversion of various compounds. In the case of HAD, the number of acids, ketones, phenols, and furans was the largest. Alcohols, aldehydes, and esters were relatively more common with VFD. With HAD, aldehydes exhibited the highest content of 457.80 µg/Kg, followed by acids, esters, phenols, ketones, furans, and alcohols. Compared with HAD, the values for esters (264.68 µg/Kg) and ketones (339.15 µg/Kg) were relatively higher with VD and VFD, respectively (Figure S2 in the Supplementary Materials). Fourteen volatile flavor compounds were simultaneously found in the different samples, among which 2-octanone (125.65–335.30 µg/Kg) presented the highest content. Ethyl caproate (72.01–118.19 µg/Kg) was the highest ester. Acetic acid (90.91–111.61 µg/Kg) and caproic acid (46.35–137.05 µg/Kg) were more prominent among the acids. Additionally, some volatile flavor compounds in diverse samples exhibited a significant difference. The aldehydes of 2-hexenal and 2,6-nonadienal largely existed in VFD samples but were not found in the HAD and VD samples. Conversely, 1-nonanal, benzaldehyde and furfuraldehyde were largely detected in the HAD and VD samples. The concentrations of 3-octenol, butyric acid, caproic acid, ethyl palmitate, methyl 4-methylvalerate, and 2,4-di-tert-butylphenol with HAD were much higher than those in VD and VFD, while 2-octanone concentrations were more abundant with VFD.

The contribution of volatile flavor compounds in mulberry was not determined by only by concentrations but also by their interaction [32]. Odor activity values (OAVs) are ratios of the concentration of volatile flavor compounds to the odor threshold (OT) in the corresponding medium and are usually applied to evaluate the aroma contribution of the compounds [33]. Table 5 shows that 8, 5, and 13 volatile flavor compounds with OAVs of ≥1 were presented in the HAD, VD, and VFD samples, respectively, indicating the important contribution of these components to the odors of mulberry. In the HAD samples, the compounds of 2,4-di-tert-butylphenol (67.15), ethyl caproate (53.73) and 1-nonanal (45.08) exhibited the highest OAVs owing to their low odor threshold values and high contents. By contrast, the compounds with relatively high OAVs in the VFD samples were 2,6-nonadienal (63.29) and 1-nonanal (39.70). These compounds might be responsible for the aroma differences observed among the three drying methods.

**Figure 8:**
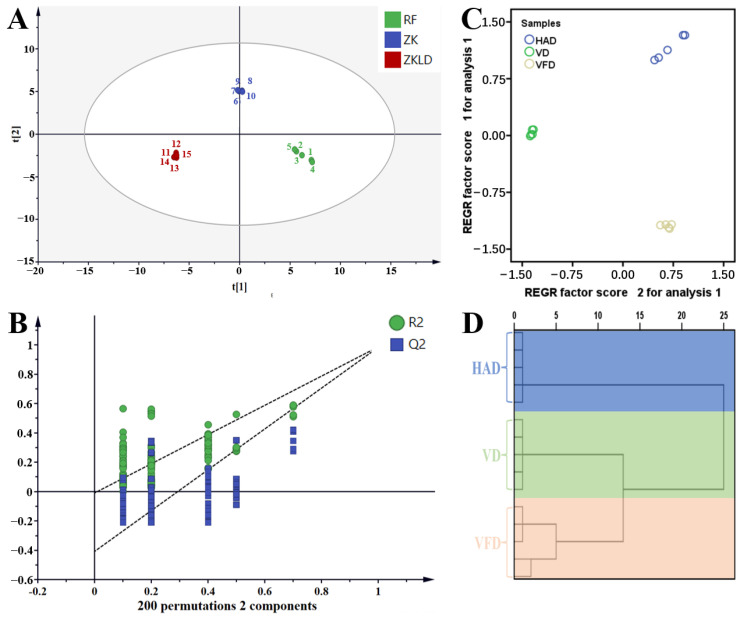
PLS-DA score plot (**A**), model validation (**B**), PCA (**C**), and HCA (**D**) of the volatile flavor compounds in mulberry samples dried by different drying methods. HAD: hot air drying. VD: vacuum drying. VFD: vacuum freeze-drying.

PLS-DA was applied to explore the flavor differences among the samples produced by the three drying methods (Figure 8A,B). A total of 20 kinds of key volatile flavor compounds with a VIP value of >1 were obtained, including methyl n-caprate, 2-phenylethanol, 3-octenol, methyl octanoate, and methyl benzoate, indicating the important roles of these components in the classification (Figure S3). Principal component analysis (PCA) and hierarchical cluster analysis (HCA) were also conducted. In the PCA analysis, the different samples were clearly separated from each other (Figure 8C). The HCA results were similar to those of the PCA. All samples were separated into three groups, and five parallel samples of each drying method were gathered into one group (Figure 8D). The above results indicate that the mulberry samples presented different volatile flavor compounds after drying with different methods.

## 4. Conclusions

In this study, the three drying techniques of HAD, VD, and VFD were applied to process fresh mulberry. The drying methods had a remarkable influence on the quality of the mulberry samples. Compared with HAD and VD, mulberry presented the highest anthocyanins (6.99 mg/g), total flavones (3.18 mg/g), and soluble sugars (2.94 mg/g), and exhibited the best DPPH· (81.2%) and ABTS^+^· (79.9%) scavenging ability with VFD. After drying with VFD, the mulberry samples showed the lowest hardness (20.5 N) and the highest brittleness (19.3 N) and exhibited the best color and sensory attributes. The volatile flavor compounds also varied according to the different drying methods; 30, 20, and 32 kinds of volatile flavor compounds were detected, respectively, in the HAD, VD, and VFD samples, among which the aldehydes, esters, and ketones were the most abundant compounds. Indeed, 2,4-di-tert-butylphenol, ethyl caproate, 1-nonanal, and 2,6-nonadienal, with their relatively higher OAVs, might be responsible for the aroma differences observed among the three drying methods. The results demonstrate that VFD is a suitable technique for the processing of mulberry, exhibiting strategic significance for promoting the development and utilization of mulberry crop resources.

## Figures and Tables

**Table 1:** Drying methods and conditions.

Drying Methods	Conditions	Time (h)
HAD	At temperature 70 °C, air velocity of 5 m/s	12
VD	At temperature 50 °C	20
VFD	Freeze-drying at temperature −50 °C, vacuum pressure 0.01 kPa	40

HAD: hot air drying; VD: vacuum drying; VFD: vacuum freeze-drying.

**Table 2:** Effects of drying methods on the phenols of mulberry. Unit: mg/kg.

Drying Methods	Gallic Acid	Protocatechuic Acid	Chlorogenic Acid	Caffeic Acid	Ferulic Acid	Cumaric Acid	Cinnamic Acid
FM	4.25 ± 0.19 ^a^	115.68 ± 4.38 ^d^	335.32 ± 6.38 ^d^	3.12 ± 0.21 ^b^	1.49 ± 0.05 ^a^	2.42 ± 0.13 ^a^	0.35 ± 0.05 ^c^
HAD	1.37 ± 0.12 ^c^	162.12 ± 3.59 ^b^	399.74 ± 7.56 ^c^	1.93 ± 0.15 ^c^	0.31 ± 0.02 ^d^	1.52 ± 0.09 ^b^	3.28 ± 0.16 ^b^
VD	1.61 ± 0.13 ^c^	189.75 ± 5.88 ^a^	711.41 ± 9.92 ^a^	3.40 ± 0.19 ^a^	0.53 ± 0.04 ^b^	1.52 ± 0.07 ^b^	3.86 ± 0.13 ^a^
VFD	3.60 ± 0.16 ^b^	122.63 ± 4.32 ^c^	421.87 ± 5.59 ^b^	1.39 ± 0.08 ^d^	0.40 ± 0.04 ^c^	0.92 ± 0.05 ^c^	0.22 ± 0.03 ^c^

Different letters represented statistically different values at *p* < 0.05.

**Table 3:** Effects of drying methods on the color of mulberry samples. FM: fresh mulberry. HAD: hot air drying. VD: vacuum drying. VFD: vacuum freeze-drying. *L*, *a*, and *b* represent blackness (0)/brightness (100), greenness (−)/redness (+), and blueness (−)/yellowness (+), respectively. Δ*E* was calculated from *L*, *a*, and *b*.

Drying Methods	*L*	*a*	*b*	Δ*E*
FM	6.52 ± 0.05 ^d^	2.13 ± 0.06 ^d^	−0.97 ± 0.06 ^d^	—
HAD	8.53 ± 0.06 ^c^	2.70 ± 0.04 ^b^	−0.82 ± 0.06 ^c^	3.20 ± 0.07 ^b^
VD	12.45 ± 0.05 ^b^	2.25 ± 0.08 ^c^	−0.45 ± 0.02 ^b^	1.18 ± 0.08 ^c^
VFD	17.17 ± 0.05 ^a^	3.83 ± 0.06 ^a^	−0.17 ± 0.01 ^a^	6.00 ± 0.03 ^a^

Different letters represented statistically different values at *p* < 0.05.

**Table 4:** Concentrations of volatile flavor compounds in dried mulberry. HAD: hot air drying. VD: vacuum drying. VFD: vacuum freeze-drying.

Species	No.	Components	Content (µg/Kg)		
			HAD	VD	VFD
Alcohols	1	Isoamylol	0 ^b^	0 ^b^	6.82 ± 0.35 ^a^
	2	Hexanol	0 ^b^	0 ^b^	6.41 ± 0.24 ^a^
	3	Isooctanol	13.02 ± 0.20 ^a^	11.97 ± 1.00 ^b^	10.92 ± 0.33 ^c^
	4	3-Octenol	13.87 ± 1.01 ^a^	5.93 ± 0.15 ^c^	10.99 ± 0.15 ^b^
	5	2-Phenylethanol	5.46 ± 0.17 ^b^	0 ^c^	6.25 ± 0.22 ^a^
	6	Terpinen-4-ol	0 ^b^	0 ^b^	4.25 ± 0.80 ^a^
Aldehydes	7	2-Hexenal	0 ^b^	0 ^b^	34.99 ± 0.55 ^a^
	8	Heptaldehyde	73.33 ± 6.53 ^a^	0 ^b^	0 ^b^
	9	Octanal	0 ^b^	0 ^b^	8.89 ± 0.54 ^a^
	10	(E)-2-Octenal	0 ^b^	0 ^b^	7.04 ± 0.92 ^a^
	11	1-Nonanal	49.59 ± 14.87 ^a^	32.82 ± 1.53 ^c^	43.67 ± 6.31 ^b^
	12	(E, E)-2,4-Nonadienal	0 ^b^	0 ^b^	5.00 ± 0.42 ^a^
	13	2,6-Nonadienal	0 ^b^	0 ^b^	50.64 ± 3.42 ^a^
	14	Caprinaldehyde	0 ^b^	0 ^b^	8.07 ± 0.96 ^a^
	15	Benzaldehyde	143.90 ± 9.63 ^a^	109.57 ± 9.17 ^b^	0 ^c^
	16	Phenylacetaldehyde	71.93 ± 3.08 ^a^	0 ^b^	0 ^b^
	17	Furfuraldehyde	121.28 ± 2.93 ^a^	17.58 ± 2.01 ^b^	0 ^c^
	18	5-Methyl furfural	15.77 ± 1.95 ^a^	0 ^b^	0 ^b^
Acids	19	Acetic acid	111.61 ± 7.32 ^a^	90.91 ± 5.48 ^b^	108.24 ± 6.62 ^a^
	20	Butyric acid	30.63 ± 5.34 ^a^	14.74 ± 1.42 ^b^	17.13 ± 1.80 ^b^
	21	2-Methyl butyric acid	0 ^b^	0 ^b^	7.93 ± 1.64 ^a^
	22	Valeric acid	18.19 ± 1.42 ^a^	0 ^b^	0 ^b^
	23	Caproic acid	137.05 ± 13.13 ^a^	51.08 ± 1.53 ^b^	46.35 ± 2.49 ^b^
	24	Octanoic acid	20.29 ± 2.26 ^a^	0 ^b^	0 ^b^
Esters	25	Methyl octanoate	16.11 ± 3.04 ^b^	35.73 ± 4.80 ^a^	14.21 ± 3.91 ^b^
	26	Methyln-nonanoate	30.92 ± 10.10 ^a^	29.09 ± 1.69 ^a^	18.08 ± 2.83 ^b^
	27	Methyl benzoate	8.66 ± 0.53 ^b^	0 ^c^	23.80 ± 1.09 ^a^
	28	Ethyl benzoate	0 ^b^	0 ^b^	11.62 ± 0.50 ^a^
	29	Ethyl caproate	118.19 ± 17.19 ^a^	111.79 ± 13.03 ^b^	72.01 ± 1.74 ^c^
	30	Octyl acetate	11.82 ± 0.38 ^a^	0 ^b^	0 ^b^
	31	Ethyl oleate	16.03 ± 0.37 ^a^	0 ^b^	0 ^b^
	32	Methyl hexadecanoate	0 ^c^	14.97 ± 1.29 ^b^	37.15 ± 2.58 ^a^
	33	Ethyl palmitate	37.03 ± 6.93 ^a^	7.52 ± 1.37 ^b^	4.66 ± 0.28 ^b^
	34	Methyl 4-methylvalerate	77.46 ± 1.81 ^a^	57.41 ± 3.96 ^c^	62.59 ± 2.80 ^b^
	35	Methyl n-caprate	0 ^b^	3.62 ± 0.12 ^a^	0 ^b^
	36	Methyl laurate	0 ^c^	4.54 ± 0.33 ^b^	13.45 ± 2.40 ^a^
	37	Methyl myristate	0 ^b^	0 ^b^	7.85 ± 1.78 ^a^
Ketones	38	2-Octanone	125.65 ± 19.78 ^c^	261.75 ± 17.59 ^b^	335.30 ± 22.56 ^a^
	39	Geranylacetone	0 ^b^	0 ^b^	3.85 ± 0.43 ^a^
	40	1,2-Cyclopentanedione	8.32 ± 0.91 ^a^	0 ^b^	0 ^b^
Phenols	41	2,4-Di-tert-butylphenol	33.57 ± 13.10 ^a^	13.43 ± 1.61 ^b^	7.69 ± 1.28 ^c^
	42	Eugenol	0 ^b^	0 ^b^	6.83 ± 0.19 ^a^
	43	Hydroquinone	167.37 ± 4.09 ^a^	0 ^b^	0 ^b^
	44	Guaiacol, 3-allyl- (6CI)	13.29 ± 1.11 ^a^	6.45 ± 0.33 ^c^	9.26 ± 0.57 ^b^
Furans	45	2-Pentylfuran	70.29 ± 12.67 ^a^	62.20 ± 5.92 ^b^	0 ^c^
	46	2-Acetylfuran	6.57 ± 0.01 ^a^	0 ^b^	0 ^b^
	47	(E)-2-(2-pentenyl) furan	8.55 ± 0.87 ^a^	0 ^b^	0 ^b^

Different letters represented statistically different values at *p* < 0.05.

**Table 5:** Odor threshold (OT) and odor activity values (OAVs) of the major volatile flavor compounds in dried mulberry. HAD: hot air drying. VD: vacuum drying. VFD: vacuum freeze-drying.

No.	Components	OT (µg/kg)	Odor Descriptor	OAVs		
				HAD	VD	VFD
1	Isoamylol	200	Alcoholic	-	-	<1
2	Hexanol	5.6	Grass, grain	-	-	1.15
3	3-Octenol	1.5	Mushroom	9.25	3.95	7.33
4	2-Phenylethanol	564.23	Floral, sweet	<1	-	<1
5	2-Hexenal	17	Ester	-	-	2.06
6	Heptaldehyde	10	Orange	7.33	-	-
7	Octanal	0.59	Lemon	-	-	15.07
8	(E)-2-Octenal	3	Nutty	-	-	2.35
9	1-Nonanal	1.1	Citrus-like, soapy	45.08	29.83	39.70
10	(E,E)-2,4-Nonadienal	1.06	Ester, floral	-	-	4.718
11	2,6-Nonadienal	0.8	Cucumber-like	-	-	63.29
12	Caprinaldehyde	3	Orange	-	-	2.69
13	Benzaldehyde	750.89	Almond	<1	<1	-
14	Phenylacetaldehyde	6.3	Floral, rose	11.42	-	-
15	Furfuraldehyde	970	Tar, wood	<1	<1	-
16	5-Methyl furfural	1110	Sweetness	<1	-	-
17	Acetic acid	22,000	Vinegar	<1	<1	<1
18	Butyric acid	2400	Sweaty, rancid	<1	<1	<1
19	Caproic acid	2600	Sweaty	<1	<1	<1
20	Octanoic acid	3000	Cheesy	<1	-	-
21	Methyl benzoate	1	Fruity	8.66	-	23.80
22	Ethyl benzoate	0.4	Floral, fruity	-	-	29.04
23	Ethyl caproate	2.2	Fruity	53.73	50.82	32.73
24	Methyl hexadecanoate	2000	Fruity	-	<1	<1
25	Ethyl palmitate	2000	Creamy	<1	<1	<1
26	Geranylacetone	60	Floral	-	-	<1
27	2,4-Di-tert-butylphenol	0.5	Camphor	67.15	26.86	15.39
28	2-Pentylfuran	5.8	Fruity	12.12	10.73	-

## Data Availability

The original contributions presented in the study are included in the article/Supplementary Material; further inquiries can be directed to the corresponding authors.

## Supplementary Materials

The following supporting information can be downloaded at: https://www.mdpi.com/article/10.3390/foods13162492/s1, Supplementary data to this article are listed in the Supplementary Materials.
