# Supplementary material for: Effect of Different Drying Techniques on the Bioactive Compounds, Antioxidant Ability, Sensory and Volatile Flavor Compounds of Mulberry
Source: Foods. 2024 Aug 8;13(16):2492. doi: 10.3390/foods13162492 (PMC11354017; doi:10.3390/foods13162492)
Supplement: Supplementary file 1 [file foods-13-02492-s001.zip › foods-3107519-supplementary.docx]

**Effect of Different Drying Techniques on the Bioactive Compounds, Antioxidant Ability, Sensory and Volatile Flavor Compounds of Mulberry**


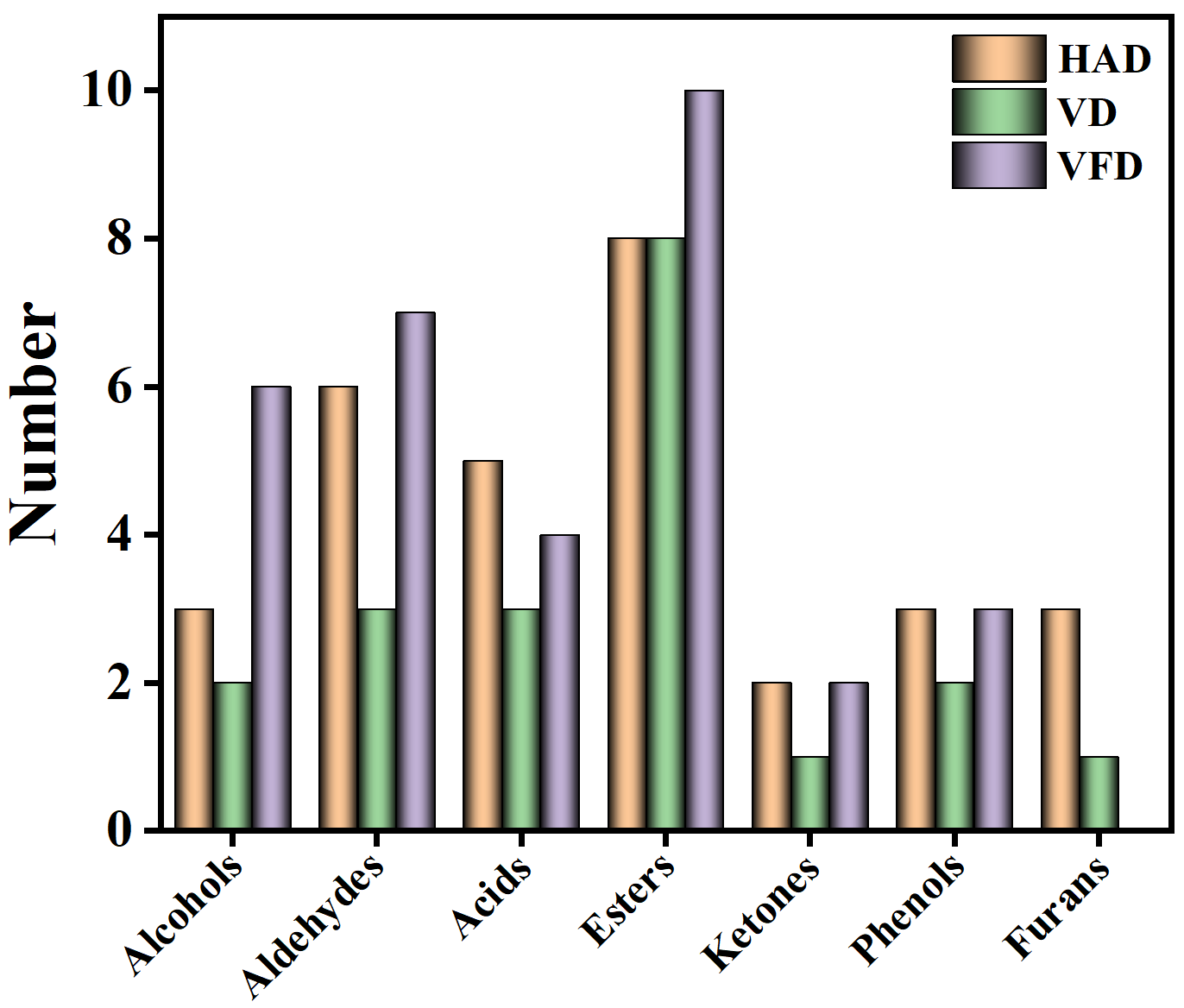


**Fig. S1**. Quantity of volatile flavor compounds dried by different methods.


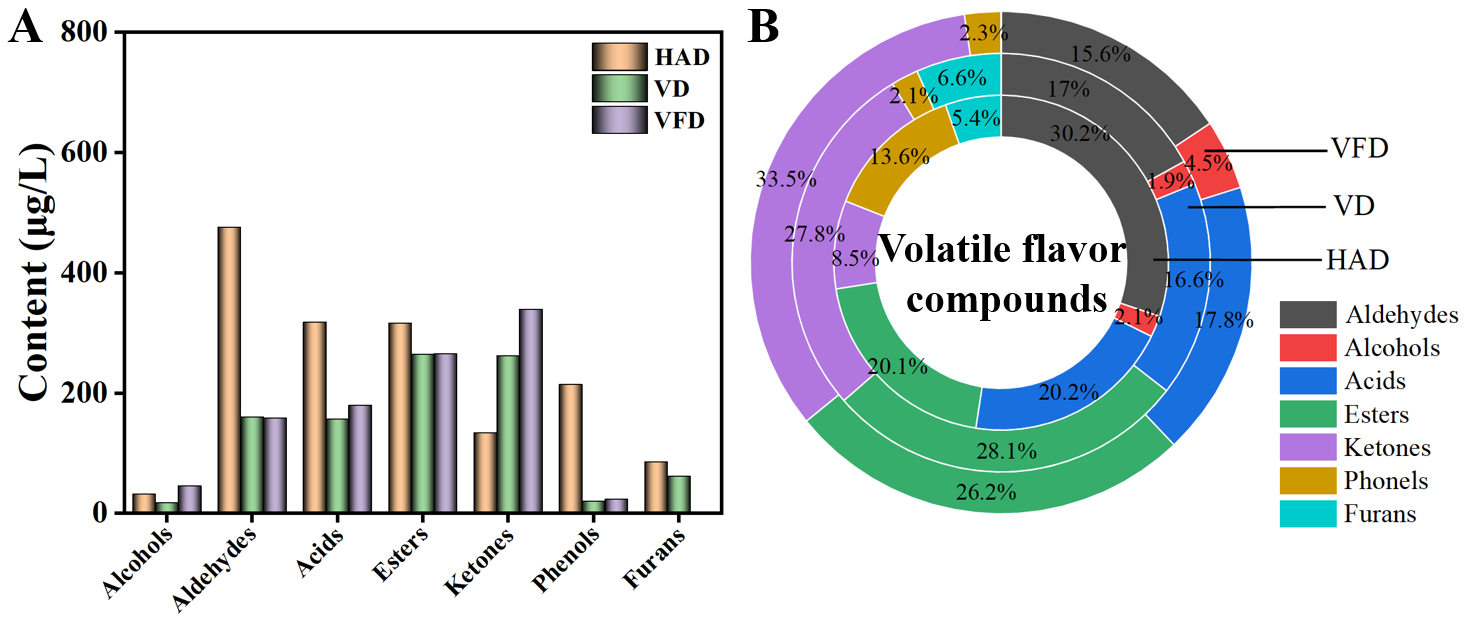


**Fig. S2**. Content (A) and proportion (B) of volatile flavor compounds dried by different methods.


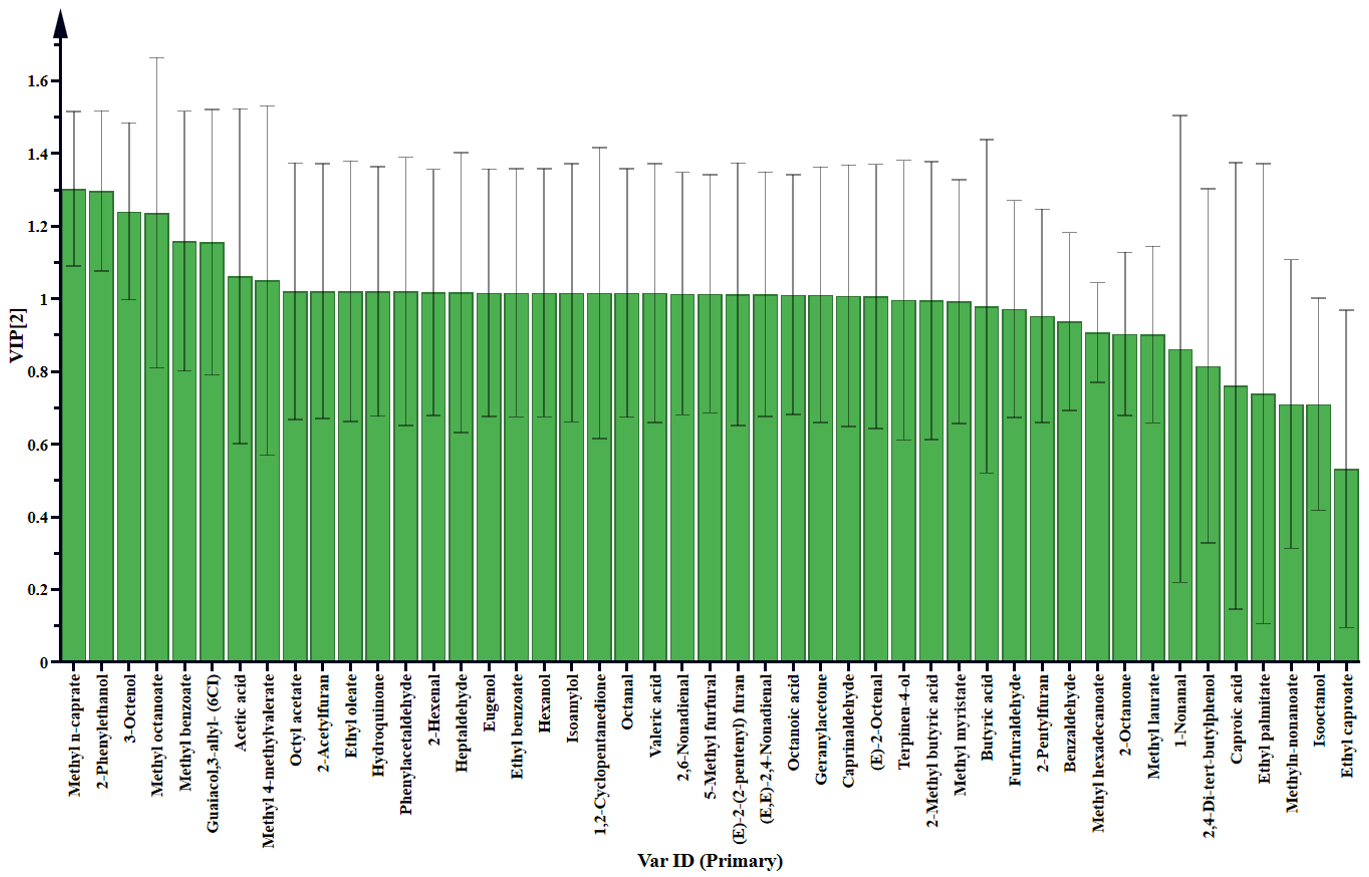


**Fig. S3**. VIP value of the key volatile flavor compounds.
